# Association between dietary intake of folate, vitamin B_6_, B_12_ & MTHFR, MTR Genotype and breast cancer risk

**DOI:** 10.12669/pjms.301.4189

**Published:** 2014

**Authors:** Zheng Weiwei, Chen Liping, Li Dequan

**Affiliations:** 1 Zheng Weiwei, General Surgery Department, The First Affiliated Hospital of Xinxiang Medical University, Weihui, China.; 2 Chen Liping, General Surgery Department, The First Affiliated Hospital of Xinxiang Medical University, Weihui, China.; 3 Li Dequan, Department of Breast Surgery, the Affiliated Tumor Hospital, Guangxi Medical University, Nanning, China.

**Keywords:** Breast cancer, Folate, MTHFR, MTR, Polymorphism, Vitamin

## Abstract

***Objective:*** we conducted a case-control study to investigate the association between dietary folate, vitamin B_6_ and vitamin B_12_ intake, MTHFR and MTR genotype, and breast cancer risk.

***Methods:*** Genotyping for MTHFR C677T and A1298C and MTR A2756G polymorphisms were performed using polymerase chain reaction-restriction fragment length polymorphism analysis (PCR-RFLP) method. The intake of folate, vitamin B_6_ and vitamin B_12_ were calculated by each food item from questionnaire.

***Results:*** Subjects with breast cancer tended to have more first-degree relatives (χ^2^**=**30.77, *P*<0.001) and have high intake of folate (*t*=2.42, *P*=0.008) and Vitamin B_6_ (*t*=2.94, *P*=0.002). Compared to the reference group, women with MTHFR 677 TT genotype and T allele had a significantly increased risk of breast cancer, with ORs (95%CI) of 1.8(1.08-2.27) and 1.39(1.02-1.92), respectively. For those who had folate intake＜450 ug/day, MTHFR 667TT genotype was associated with a higher risk of breast cancer (OR=2.45, 95% CI=1.09-5.82, P=0.02). Similarly, subjects with Vitamin B_6_ intake＜0.84 mg/day and MTHFR 667T allele genotype was correlated with a marginally increased risk of breast cancer. A significant interaction was observed between MTHFR C667T polymorphism and folate intake on the risk of breast cancer (*P* for interaction was 0.025).

***Conclusion:*** This case-control study found a significant association between MTHFR C667T polymorphism, folate intake and vitamin B_6_ and breast cancer risk, and a significant interaction was observed between MTHFR C667T polymorphism and folate intake on the risk of breast cancer.

## INTRODUCTION

Breast cancer is the most common malignancy in women worldwide, and it has become the second leading cause of death in Chinese females.^1^ The etiology of breast cancer is complicated and not well clarified, but recent efforts have indicated that a complex combination of genetic and environmental factors have a role in the development of breast cancer.^2^ Deficiency of nutrients, such as vitamins and microelements, were observed to be correlated with breast cancer, but a high intake of fruits and vegetables are considered to have a protective role in the development of breast cancer.^3^

Folate and other methyl-related B vitamins are necessary for DNA synthesis, integrity and stability.^4^ Deficiency of folate and methyl-related B vitamins could induce defective DNA repair and chronmosomal fragile site expression, and thus cause chromosomal breaks and micronucleus formation.^5^ These functions could play an important role in the development of various cancers. ^6^^-^^10^ The enzyme 5,10-methylenetetrahydrofolate reductase (MTHFR) irreversibly catalyzes the conversion of 5,10-methylenetetrahydrofolate (5,10-methylene THF) to 5-methyltetrahydrofolate (5-methyl THF), play an important role in folate metabolism, and the polymorphisms in folate-metabolizing genes may influence the function of synthesis in DNA repair.^11^ Two common genetic polymorphisms in MTHFR (C677T and A1298C) and one in Methionine synthase (MTR) (MTR A2756G) were widely studies, and found the genetic variations of MTHFR and MTR are associated with increased or reduced risk of breast cancer.^4^^,^^12^^-^^15^

Previous studies have suggested an inverse association between high consumption of folate intake and methyl-related B vitamins and risk of breast cancer in different populations.^15^^, ^^16^ However, the results of studies regarding the protective effects of folate and B vitamins intake against breast cancer risk remains inconsistent.^15^^-^^17^ Therefore, we conducted a case-control study to investigate the association between dietary folate, vitamin B_6_ and vitamin B_12_ intake, MTHFR and MTR genotype, and breast cancer risk. 

## METHODS

***Subjects***: A total of 325 cases with breast cancer who were newly diagnosed and histologically confirmed were enrolled at Xinxiang Medical College and Guangxi Medical University from March 2009 to November 2011. Three hundred thirty eligible controls who entered the hospital for health check-ups during the same period were enrolled into control group. The protocol of our study was approved by the institutional review board at Guangxi Medical University and Xinxiang Medical College, and conducted in accordance with the Declaration of Helsinki. Written informed consent was collected from all patients.

A self-designed questionnaire was used to collect the clinical and demographic characteristics, such as age, age at menarche, age of menopausal status, family history of cancer, and dietary intake habits with 62 food terms. The intake of folate, vitamin B_6_ and vitamin B_12_ were calculated by each food item from questionnaire.

***Genotype of polymorphisms: ***Peripheral blood samples were obtained from each case and control subject, and stored at -80℃ until analysis. Genomic DNA samples were extracted using TIANamp blood DNA kit (Tiangen Biotech, Beijing, China) in accordance with the manufacturer’s protocol. Genotyping for MTHFR C677T and A1298C and MTR A2756G polymorphisms were performed using polymerase chain reaction-restriction fragment length polymorphism analysis (PCR-RFLP) method. Primer and probes of MTHFR C677T and A1298C and MTR A2756G were designed by Sequenom® Assay Design, Version 3.1 software (Sequenom®). The digested PCR products were separated on 3% agarose gel electrophoresis, stained by ethidium bromide and visualized by UV transilluminator. For quality control, 10% of cases and controls were randomly selected, and repeat analysis was conducted to verify the reproducibility. The results were 100% concordant.

***Statistical Analysis: ***Continuous variables and Categorical variables were presented as mean ± SD and *n *(%) of subjects, respectively, and analyzed using the independent-samples *t*-test and χ^2^-test, respectively. The association between dietary folate, vitamin B_6_ and vitamin B_12_ intake, MTHFR and MTR genotype, and breast cancer risk were assessed by unconditional logistical regression model with Odds ratios (ORs) and 95% confidence intervals (CIs). Stratified analyses were used to evaluate the potential modifying effect of modifying effect of folate intake, vitamin B_6_ and vitamin B_12 _on breast cancer risk with MTHFR genotypes. All analyses were performed by using Stata version 8 (Stata, College Station, TX). All *P*-values were two sided, and a  *P*-value <0.05 was considered statistically significant.

## RESULTS

Of 325 eligible breast cancer cases, 296 patients agreed to participate into our study, with a participation rate of 91.08%. Three hundred thirty controls were enrolled into our study, and 306 of them agreed to join this study (92.73%) (Table-I). Subjects with breast cancer tended to have more first-degree relatives (χ^2^**=**30.77, *P*<0.001) and have high intake of folate (*t*=2.42, *P*=0.008) and Vitamin B_6_ (*t*=2.94, *P*=0.002). 

**Table-I:** Characteristics of breast cancer and control subjects

	*Cases* *N=* *296*	*%*	*Controls* *N=* *306*	*%*	*t or χ* ^2^	*P-value*
Age at enrollment (mean±SD), years	48.3±7.4		47.5±8.3		1.25	0.11
<45	120	40.6	136			
≥45	176	59.4	170		0.94	0.33
Age at menarche, years						
<13	160	54.1	152			
≥13	135	45.9	154		1.25	0.26
Menopausal status						
Premenopausal	137	46.2	125	39.7	1.81	0.18
Postmenopausal	159	53.8	181	60.3		
Breast cancer in first-degree relative						
No	265	89.4	305	99.5	30.77	<0.001
Yes	31	10.6	1	0.5		
Folate intake, ug/day	496.7±108.5		517.6±103.7		2.42	0.008
Vitamin B_6_, mg/day	0.82±0.26		0.88±0.24		2.94	0.002
Vitamin B_12_, ug/day	7.1±5.1		7.7±4.2		1.58	0.06

The genotype distributions of MTHFR C677T and A1298C and MTR A2756G were in line with Hardy-Weinberg equilibrium in the control group. Compared to the reference group, women with MTHFR 677 TT genotype and T allele had a significantly increased risk of breast cancer, with ORs (95%CI) of 1.8(1.08-2.27) and 1.39(1.02-1.92), respectively (Table-II). But there was no statistically significant association between MTHFR A1298C and MTR A2756G polymorphisms and risk of breast cancer. 

**Table-II:** Association of MTHFR C677T and A1298C genotypes with breast cancer risk

*MTHFR C677T*	*Cases*		*Controls*		*P value*	*OR(95% CI)*
	*N=* *296*		*N=* *306*			
CC	156	52.70	185	60.46	-	1.0(Ref.)
CT	97	32.77	93	30.39	0.3	1.23(0.86-1.79)
TT	44	14.86	28	9.15	0.02	1.87(1.08-2.27)
CT+TT	140	47.30	121	39.54	0.13	1.39(1.02-1.92)
MTHFR A1298C						
AA	135	45.61	151	49.35	-	1.0(Ref.)
AC	129	43.58	130	42.48	0.7	1.11(0.78-1.58)
CC	32	10.81	25	8.17	0.64	1.43(0.78-2.66)
AC+CC	161	54.39	155	50.65	0.63	1.16(0.83-1.62)
MTR A2756G						
AA	149	50.34	176	57.52	-	1.0(Ref.)
AG	110	37.16	103	33.66	0.02	1.28(0.88-1.81)
GG	37	12.50	37	12.09	0.45	1.18(0.69-2.03)
AG+GG	147	49.66	140	45.75	0.02	1.26(0.91-1.73)

1. Adjusted for age, menopausal status, folate and Vitamin B_6_ intake

The associations of MTHFR C667T polymorphism and intake of folate and Vitamin B_6_ with breast cancer risk are presented in Table-III. For those who had folate intake＜450 ug/day, MTHFR 667TT genotype was associated with a higher risk of breast cancer (OR=2.45, 95% CI=1.09-5.82, P=0.02), and statistically significant association disappeared among individuals with folate intake≥500 mg/day. Similarly, subjects with Vitamin B_6_ intake＜0.84 mg/day and MTHFR 667T allele genotype was correlated with a marginally increased risk of breast cancer. A significant interaction was observed between MTHFR C667T polymorphism and folate intake on the risk of breast cancer (*P* for interaction was 0.025).

**Table-III:** Interaction between folate and Vitamin B_6_ intake and MTHFR C667T polymorphism for breast cancer risk

*Dietary intake*	*MTHFR C667T*	*Cases*	*%*	*Controls*	*%*	*P value*	*OR(95% CI)*
							
Folate intake＜450 ug/day	CC	82	48.24	79	58.09	-	1.0(Ref.)
	TT	28	16.47	11	8.09	0.02	2.45(1.09-5.82)
	CT+TT	88	51.76	57	41.91	0.09	1.50(0.94-2.42)
Folate intake≥500 mg/day	CC	74	58.73	106	62.35	-	1.0(Ref.)
	TT	16	12.70	17	10.00	0.43	1.36(0.62-3.08)
	CT+TT	53	42.06	64	37.65	0.48	1.19(0.74-1.96)
Vitamin B_6_＜0.84 mg/day	CC	82	50.93	77	57.46	-	1.0(Ref.)
	TT	23	14.29	13	9.70	0.18	1.66(0.74-3.83)
	CT+TT	79	49.07	47	42.54	0.04	1.63(0.98-2.62)
Vitamin B_6_≥0.84 mg/day	CC	74	54.81	108	62.79	-	1.0(Ref.)
	TT	21	15.56	15	8.72	0.07	2.02(0.92-4.55)
	CT+TT	62	45.93	64	37.21	0.14	1.42(0.87-2.30)

1. Adjusted for age and menopausal status

## DISCUSSION

In the present study, we found high intake of folate and Vitamin B_6_ was associated with a significantly increased risk of breast cancer, and MTHFR 677TT genotype was correlated with increased risk of breast cancer. In addition, a significant interaction was observed between MTHFR C667T polymorphism and folate intake on the risk of breast cancer. However, we did not find statistically significant association between the MTHFR A1298C and MTR A2756G polymorphisms and breast cancer risk. 

It is reported that folate plays a protective role in the development of cancer before preneoplastic lesion, and an inverse association was found between intake of folate and tumorigenesis.^17^^-^^19^ Although the high intake of folate may be beneficial in populations who are deficient of this nutrient, high intake of folate may provide further benefit or harmful for women with already-sufficient levels.^20^ In our study, we observed that high folate intake contributes to prevent the development of tumor. However, previous study indicated that an increased risk of breast cancer was found in premenopausal women with high folate intake,^4^ and another study reported no significant association between dietary intake of folate and breast cancer risk in Japanese women.^4^ Folate levels may play a protective role in breast cancer risk among those with low level of folate, but contribute to tumorigenesis in those who have been already sufficient in foalte intake group.

Moreover, previous two studies indicated that high intake of vitamin B_6_ had an association with a decreased risk of breast cancer in Chinese and Brazilian female populations.^4^^,^^21^ However, Lin et al. conducted a case-control study with 848 cases and 848 controls, and reported folate, vitamin B_6_, and vitamin B_12_ may confer litter or no reduction in overall risk of developing breast cancer.^17^ The inconsistency of these studies may be induced by differences in ethnicities, source of control subjects, sample size and etc. Further their confirmation of existing findings is still needed in future studies.

Our study found a significant association between MTHFR C677T polymorphism and breast cancer risk, and MTHFR C677T genetic variation has interaction with vitamin B_6_. Our findings are consistent with previous studies.^22^^-^^24^ A meta-analysis reported no overall significant or reverse association between MTHFR C677T polymorphism and breast cancer risk, or between the MTHFR A1298C polymorphism and risk.^25^ Ma et al. also reported the lack of association between MTHFR C677T polymorphism and breast cancer risk in Brazilian women.^4^ Another meta-analysis with pooling 37 studies indicated that MTHFR 677TT variant genotype was associated with icnreased breast cancer risk. Because these studies were conducted in different populations, direct comparisons between them are difficult. The discrepancies of these studies may be due to differences in variant frequencies between races.

In summary, this case-control study found a significant association between MTHFR C667T polymorphism, folate intake and vitamin B_6_ and breast cancer risk, and a significant interaction was observed between MTHFR C667T polymorphism and folate intake on the risk of breast cancer. Our finding indicated that folate and other methyl-related B vitamins have a role in developing breast cancer. Further multi-center studies are needed to elucidate the etiology of breast cancer. 

## Notes

***Acknowledgements: ***We thank Xinxiang Medical College (132102310163) for financial assistance for this study.
